# Is There a Pathologic Running Motion Associated with Running-Related Injuries? A Methodological Study Using a Motion Analysis System Without Sensors

**DOI:** 10.3390/medicina60081249

**Published:** 2024-07-31

**Authors:** Hyok Woo Nam, Jae Hyuk Yang, Seul Gi Park, Hye Chang Rhim, Hong Jin Kim

**Affiliations:** 1 Nam’s Orthopedic Running Clinic, 494, Yongmasan-ro, Jungnang-gu, Seoul 02182, Republic of Korea; 2 Department of Orthopedic Surgery, Korea University Anam Hospital, College of Medicine, Korea University, Seoul 02841, Republic of Korea; kuspine@korea.ac.kr (J.H.Y.); qkrtmfrl56@gmail.com (S.G.P.); 3 Department of Physical Medicine and Rehabilitation, Harvard Medical School, Spaulding Rehabilitation Hospital, Boston, MA 02141, USA; hrhim@mgh.harvard.edu; 4 Department of Orthopedic Surgery, Inje University Sanggye Paik Hospital, Seoul 01757, Republic of Korea; hongjin0925@naver.com; 5 Department of Orthopedic Surgery, Gyeong-in Regional Military Manpower Administration, Seoul 16440, Republic of Korea

**Keywords:** sports injury, running-related injury, posture, pathological movement, motion analysis

## Abstract

(1) *Background and objectives:* Running-related injuries (RRIs) are commonly attributed to improper running posture and overuse. This study aims to analyze the running motions of individuals with and without RRIs using a sensor-free method, which offers a user-friendly and straightforward approach. (2) *Materials and Methods:* A total of 155 runners were divided into two groups: the normal runner group (runners who had never been injured, n = 50) and the RRI group (runners who had experience at least one injury while running, n = 105). The forward head posture (FHP), trunk lean, hip rotation, horizontal movement of the center of gravity (COG), vertical movement of the COG, pelvic rotation, hip hike, and type of strike were measured for posture analysis. (3) *Results:* We found that the left–right balance of the pelvis and the spinal posture during running were associated with RRIs. The difference in hip hike and FHP emerged as key predictors of running-related musculoskeletal injury occurrence from our logistic regression analysis. (4) *Conclusions:* Identifying pathological movements in runners through running motion analysis without the use of sensors can be instrumental in the prevention and treatment of RRIs.

## 1. Introduction

Running-related injuries (RRIs) are prevalent, with incidence rates ranging from 19% to 92%, depending on the definition [1]. Various potential causes of RRIs have been reported in the literature, from demographic factors including body mass index (BMI), past injury history of RRIs, being female, and age to running-related factors including changes in running distance and speed, training frequency and intensity, consistency of training regimen, competitive running, step length, and running experience [2]. In particular, medial tibial stress syndrome (MTSS), patellofemoral pain syndrome (PFP), iliotibial band syndrome (ITBS), and Achilles’ tendinopathy (AT) were classified as RRIs, which are closely associated with pathologic running mechanics [1]. Specifically, PFP and ITBS are frequently linked with abnormal hip adduction, while MTSS and PFP are often observed alongside an increased internal rotation of the hip joint [3,4]. Additionally, the hyperpronation of the foot is a known contributor to MTSS and AT [5,6,7].

Each runner exhibits a distinct movement pattern characterized by a unique balance in various directions in joint movements [8]. It is rare for runners to maintain a proper spinal posture, balanced pelvis and hips, and well-aligned knee and ankle movements, all of which are essential for preventing RRIs and sustaining overall body health and physical function [9,10]. However, the imbalance of running mechanics in each runner can result in abnormal movements [11]. Thus, the repetition of such abnormal movements may lead to the development of RRIs [10,11].

To elucidate the running-related factors originating from abnormal running biomechanics, several researchers tried to analyze the running movements and mechanics using the motion analysis system [12,13]. Traditional instruments that utilize sensors have been applied, but they are relatively difficult to experiment with and hard to use with various runners [14]. By considering evident changes in running patterns based on personal analysis, activities, and the ability to adapt running exercises in different types of environments, it is essential to measure pathological running motion by using a motion analysis system without sensors [14].

Although biomechanical changes have been observed in several studies, they are unclear due to the variability of the range of motion [15,16,17]. Furthermore, there are few studies measuring pathological running motion using a motion analysis system free from sensors. Recently, several commercialized 3D motion-capturing systems without sensors have been introduced; they automatically recognize joint landmarks and measure the gait motions with a rear-facing camera without a sensor, which is an easy-to-use and straightforward approach. Given the limited research on the utilization of a commercialized 3D motion-capturing system, we tried to investigate pathologic running motion in runners with RRIs. Therefore, this retrospective comparative study aimed at elucidating whether abnormal pelvic movements and improper spinal tilting are influenced by running style and strike type between runners with and without RRIs. Specifically, we analyzed differences in upper-body posture, center of gravity, and pelvic movement using a motion analysis system without sensors.

## 2. Materials and Methods

This retrospective comparative study was conducted after review and approval by the Institutional Review Board.

### 2.1. Study Design, Participants and the Inclusion and Exclusion Criteria

**Figure 1:**
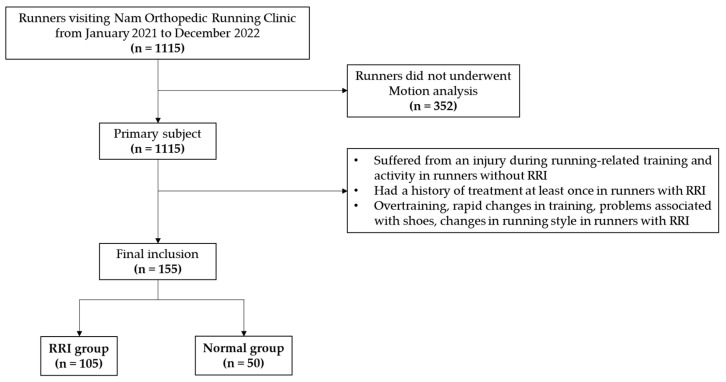
A flowchart of this study.

This study included 1115 runners who visited Nam Orthopedic Running Clinic from January 2021 to December 2022. Among them (the participants, consisting of novice, recreational, and elite or professional runners), there were a total of 982 patients with RRIs and 133 runners without RRIs. Of these, 763 runners underwent running motion analysis using a motion analysis system (30 Hz, Exbody, Seoul, Republic of Korea). They were selected as the primary study subjects. The normal runner group (n = 133) was defined as runners who had never suffered from an injury during running-related training and activity. The RRI group was composed of runners who had been treated for an RRI at least once. Of the 763 runners who were selected as the primary study subjects, the RRI group (n = 219) excluded cases in which the cause of the injury was relatively clear, such as overtraining, rapid changes in training (more than 30% change in running speed, number of runs, distance, and speed per week), problems associated with shoes (such as damage to the sole of the shoe, shoes that did not fit the foot shape, and changes in heel-to-toe drop), and changes in running style (injuries that occurred after changing the running style within one week). Individuals with a history of previous trauma or other surgical conditions, in addition to RRIs, were excluded from the study. Running motion analysis for the RRI group (n = 763) was performed when the acute pain was treated, and when normal running was deemed possible. A total of 155 runners met the inclusion criteria (post-hoc power: 0.97). Of these, 105 runners experienced an injury while running (RRI group), while 50 runners were normal (normal runner group). The detailed study flowchart is presented in Figure 1.

**Figure 2:**
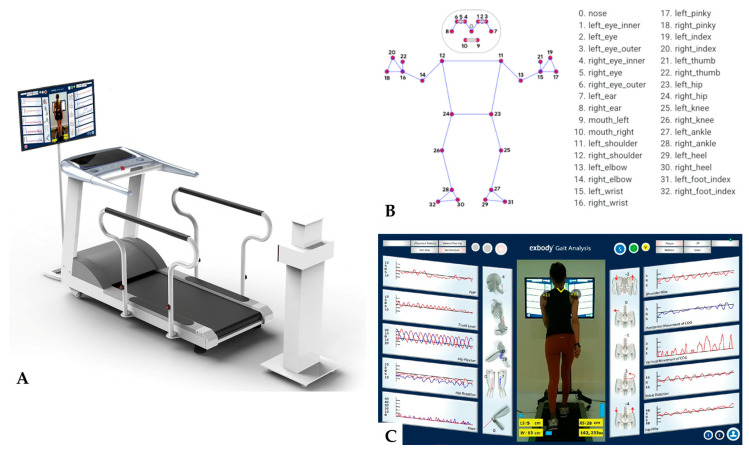
Running motion analysis was performed using a motion analysis system (30 Hz, Exbody, Republic of Korea) with a rear-facing camera without a sensor. (**A**) Equipment for the motion analysis system. (**B**) Landmarks for detecting in runners. (**C**) Gait analysis observed the running posture from head posture to leg motion. The red line means left-sided and blue line means right-sided.

All runners were asked to complete a self-administered questionnaire about RRI. The data from the questionnaire on their intrinsic running characteristics included running experience, weekly distance, average running speed, distance practiced, and running course, among other factors. Anthropometric measurements, including weight and height, were taken. Body composition was analyzed using the InBody 270 machine (InBody Co., Seoul, Republic of Korea) through an in-body examination. Radiographs and ultrasounds were conducted for each injury, and the affected areas were evaluated by an orthopedic surgeon. Then, the following variables were bilaterally measured among normal runners and runners with RRIs by the running motion analysis: forward head posture (FHP), trunk lean, hip rotation, horizontal movement of the center of gravity (COG), vertical movement of COG, pelvic rotation, and hip hike. Running motion analysis was performed using a motion analysis system (30 Hz, Exbody, Republic of Korea) with a rear-facing camera without sensors (Figure 2). From this system, we quickly evaluated the gait motion of individuals training on the treadmill in real time without sensors.

### 2.2. The Data Collection from Questionnaire

The questionnaire included detailed questions about running experience; running distance per week; average running speed; distance practiced; running course; running terrain; medical history; previous history of injury; participation in competitions; and recent changes in the training schedule, footwear, training regimen, weight, running style, and perceived type of strike. In the survey, the novice runner group was defined as runners who had been running for less than six months and had never competed in a race. The recreational group was defined as runners who had been running regularly for at least six months twice to thrice a week and had competed in a race. Elite or pro runners were defined as runners who had been running professionally since they were young.

### 2.3. Running Motion Analysis

Runners who underwent the running motion analysis were asked to stand on a treadmill before running to measure their body reference alignment. After automatically recognizing body joint markers using an instrument, the runners moved from a walking pace to their normal running speed. Before starting the measurement, the runners were asked to run for 2 min while using the rearview camera to analyze their running motion, ensuring that it felt the same as their normal running motion. The rearview camera allowed us to obtain measurements of the FHP, trunk lean, hip rotation, horizontal movement of the COG, vertical movement of COG, pelvic rotation, the hip hike. Although the measurement display was in 2D, the data were analyzed in a 3D format. This design allows for real-time motion tracking across the *X*, *Y*, and *Z* axes. Based on ‘Motion Tracking’ technology, the ‘Pose Estimation’ method is employed to track and estimate whole-body landmarks, including upper and lower limbs (such as shoulders and elbows) in each frame, thereby collecting data [18]. The collected data are represented as coordinates in decimal form and are still raw, unprocessed data. Therefore, they are converted through ‘Mapping’ to be usable. During ‘Mapping’, at least one reference point is established. Based on this reference, the moving points are expressed as angles using our proprietary logic, and the final data (movement distance and angle) are calculated.

Additionally, two side-view cameras were used, with their positions illustrated in Figure 1; they recorded whether the part of the foot that contacted the ground was both the midfoot and forefoot or the rearfoot alone (type of strike). We classified midfoot and forefoot in the same category based on previous studies that included runners with midfoot strike patterns in the forefoot group since the biomechanical aspects of the forefoot strike and midfoot strike closely resemble one another [19].

### 2.4. Statistical Analysis

Statistical analyses were performed using the Statistical Package for Social Sciences software (version 25, IBM Corp., Armonk, NY, USA). Univariate analysis was performed to compare the demographic data and the measurements acquired from the motion analysis system between the injured runners and the normal group. The continuous variables were compared using the independent t-test after confirming the normal distribution through the Kolmogorov–Smirnov test. The categorical variables were compared using the chi-square test or Fisher’s exact test, appropriately. Logistic regression analysis was performed using the variables that were significantly different between the injured runners and the normal group to investigate the association between the variable and RRIs. The intraclass correlation coefficient (ICC) for measured outcomes was preliminary measured using a motion analysis system with a rear-facing camera without a sensor with 15 normal individuals, which was calculated by Cronbach’s alpha. A *p* value less than 0.05 was considered significant.

## 3. Results

### 3.1. Demographic Data and Running Analysis Measurements

The average age of the runners was 38.9. Additionally, there were 110 male and 45 female participants. The average height was 174 cm, while the average weight was 66.7 kg. The average BMI was 22.77. The average distance run per week was 33.3 km/week. Of the 155 total participants, 110 (71%) were male and 45 (29%) were female. In the normal runner group consisting of 50 members, 33 (66%) were male and 17 (34%) were female. In the RRI group, 77 (73.3%) were male and 28 (36.7%) were female.

Motion analysis using the rear camera showed that the average angle of FHP was 8.19°; the average angle of the trunk lean was 6.19°; the mean of hip rotation RT and LT were 7.79° and 5.41°, respectively; the mean horizontal movement of the COG RT and LT was respectively 1.69° and 2.46°; the mean value of the vertical movement of the COG was 2.78°; the mean pelvic rotation RT and LT was 5.84° and 7.97°, respectively; the mean hip hike RT and LT was, respectively, 3.08° and 0.74°; and the mean value of the difference in hip rotation RT-LT, horizontal movement of the COG RT-LT, pelvic rotation RT-LT, and hip hike RT-LT were, respectively, 2.37°, −0.77°, −2.12°, and 2.33°. The demographic data and running analysis measurements of the total enrolled runners are listed in detail in Table 1. 

When comparing the normal runner group with the RRI group, no differences were observed between the groups for age (*p* = 0.347), weight (*p* = 0.111), height (*p* = 0.344), BMI (*p* = 0.327), and running distance (*p* = 0.204). However, statistically significant differences were observed in several parameters: FHP, horizontal movement of the center of gravity (COG) on the left (LT), hip hike on the right (RT), hip hike on the left (LT), the difference in horizontal movement of the COG (RT-LT) and the difference in hip hike (RT-LT) (all Ps < 0.05). Detailed statistical analysis results are presented in Table 2.

### 3.2. The Type of Foot Strike during Running

When the type of foot strike during running was analyzed using the lateral camera, there were 104 rearfoot strikes, with a total of 155 and 32 mid/forefoot strikes, with a total of 155. The rate of rearfoot strikes alone was 67%, while that of mid/forefoot strikes was 32.9%. When the injured runners were compared to the normal runners, there was no significant difference between the two groups (*p* = 0.351) (Table 3).

### 3.3. Relationship between Running Experience and RRI

There was a statistical difference in RRIs between the beginner and recreational group (*p* = 0.033). Detailed information is presented in Table 4.

### 3.4. Logistic Regression Analysis between Running Motion Analysis Items and RRI

FHP and difference of hip hike (RT-LT) were statistically significantly correlated with injury occurrence during running (all *p* < 0.001) (Table 5). These results demonstrated that by conducting running motion analysis using a motion analysis system, using only the rearview camera, FHP (turtle neck) and the difference in hip hike (RT-LT) can be used to recognize RRIs during running and identify injured runners.

### 3.5. ICC for Measured Outcomes

The ICC values for all measured outcomes in normal individuals were higher than 0.6, indicating proper reliability (questionable, acceptable, good, and excellent reliability). Detailed information on ICC values is presented in Table 6. The ICC for measured outcomes showed a more than acceptable range of reliability (ICC more than 0.6) (Table 6).

## 4. Discussion

This study aims to analyze the running motions of runners with and without RRIs using only a rear-facing camera without utilizing sensors, as the method offers a user-friendly and straightforward approach. Specifically, we examined differences in upper-body posture, center of gravity, and pelvic movement using a motion analysis system that does not rely on sensors. Based on our study, it was observed that FHP and the difference in the hip hike were significantly elevated in the RRI group compared to the normal runner group, as measured using the motion analysis system. Notably, the logistic regression analysis demonstrated that the difference in hip hike was a significant predictor of injury occurrence, yielding a high odds ratio of 17.168, as shown in Table 3. Among the various running-motion test variables, this difference in hip hike was the most effective in distinguishing the RRI group from the normal runner group. Hip hike is defined as the tilt of the pelvis to the left and right, measured by the rear camera of the motion analysis system [20]. Therefore, our study suggested that the difference in hip hike is an important factor for RRIs, which was demonstrated by our motion analysis system free from sensors.

**Figure 3:**
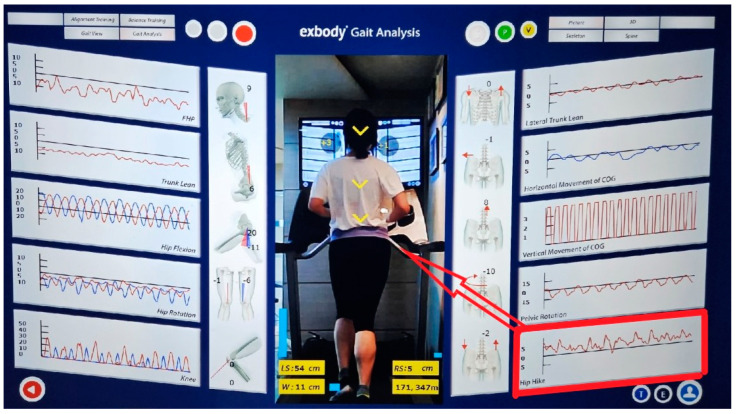
Hip hike refers to the left and right tilt of the pelvis measured by the rear camera of the motion analysis system (30 Hz, Exbody, Seoul, Republic of Korea). The difference in hip hike (RT-LT) refers to the difference between the right and left hip hikes. In the normal runner group, the difference between the difference in hip hike (RT-LT) and pelvic tilt was less than 1°. However, in the RRI group, the difference in hip hike (RT-LT) showed a difference of more than 3°. The red line means left-sided and blue line means right-sided.

The phrase ‘the difference in hip hike’ refers to the difference between the right and left hip hikes (Figure 3). In the normal runner group, the difference between the hip hike and pelvic tilt was less than 1°. However, in the RRI group, the difference in hip hike showed a difference of more than 3°. Therefore, clinicians should focus on hip hike values in runners to potentially prevent RRIs.

The observed variance in hip hike may be attributed to several factors, including the diminished strength of the gluteus medius muscle on the left side, leading to contralateral pelvic drop (CPD) [21]. Additional contributing factors include disparities in hip flexibility and range of motion, deliberate pelvic elevation to facilitate toe clearance, and asymmetries in the strength of the lower extremities between the left and right sides [16]. Individuals exhibiting this hip hike discrepancy typically showed a reduced range of motion and flexibility on the left side [20]. Furthermore, the left gluteus medius was consistently weaker compared to the right during activities such as single-leg standing, lunges, and stair-climbing exercises [22]. As a compensatory mechanism, there was an observed upward movement of the right pelvis and pelvic sway during initial contact to ensure toe clearance. Notably, in instances where the left gluteus medius was weak, the frequency of right hip hike occurrences surpassed that of the left. Although there may be cases where the right gluteus medius is weaker in individuals, our findings predominantly indicated a higher prevalence of left gluteus medius weakness. Thus, it is plausible that the pattern of hip hike could be reversed in such scenarios, leading to the occurrence of the left hip hike.

Previous studies have identified that CPD is an important kinematic parameter related to RRI [21,23]. Weakness in the ipsilateral hip abductors, particularly the gluteus medius, can potentially result in CPD [23]. Increased CPD contributes to the etiology of ITBS by increasing the tension and pressure between the iliotibial band and the lateral femoral condyle [24]. Concurrently, tension in the iliotibial band can displace the patella laterally, and then increases patellofemoral joint stress, thereby leading to PFP [3,25]. Elevated CPD shifts the center of ground-reaction force to the medial aspect of the patella, thus increasing internal rotation and the bending forces in the shin and foot. These findings have also been reported to be associated with MTSS and AT [11,16,26].

This study identified that differences in hip hikes were primarily associated with higher right and left hip hikes. A hip hike disparity of three or more degrees between the right and left sides correlated with RRI. Physical assessments revealed significant weaknesses in the left hip abductors and gluteus medius muscles among RRI patients. It was unusual for hip hikes to increase and CPD to occur on opposite sides of the body. Runners avoided initial foot contact on the side with the weaker hip abductor. The compensatory mechanism to address the instability involved an increased contralateral pelvic rise. In contrast to previous research, this study utilized a simplified assessment method to measure hip hikes and identify pelvic imbalance. This approach allows for easier detection of pelvic discrepancies, making it more accessible for routine screenings. The study’s results suggest that screening for pelvic imbalances, such as CPD, could serve as a valuable tool for identifying runners at higher risk of injuries. However, Further investigation into the causes of hip hike elevation and CPD discrepancies from multiple perspectives is warranted [12,22]. Based on the study’s results, it is recommended that both novice and professional runners recovering from injury should engage in strengthening exercises to build strength and balance in the hip abductors through activities such as lunges and squats. Additionally, runners should aim to develop a stable and balanced pelvis and gradually increase their running frequency and intensity to avoid unstable pelvic movements.

The appropriate spinal posture during running remains a subject of debate. Increased forward trunk lean has been associated with knee extension and ankle dorsiflexion at initial contact, which Bramah et al. noted increases the risk of RRI by shifting the center of mass away from the point of initial contact [12]. Conversely, the absence of forward trunk lean can make it challenging to generate the necessary repulsive force or momentum for push-off. Therefore, the optimal level of spinal tilt for efficient running with minimal injuries remains unclear.

The mean angle of trunk lean observed in this study was 6.19°, with no significant difference between the normal and RRI groups. However, the FHP measured 5.5° in the normal runner group compared to 9.47° in the RRI group, with the difference being statistically significant. This finding suggests that trunk flexion is more prevalent in the RRI group, implying a potential association between trunk flexion and RRI. Trunk flexion, characterized by greater cervical spine flexion compared to the thoracolumbar spine, contrasts with forward trunk lean, where both the cervical and thoracolumbar spine tilt together. Running with trunk flexion shifts the weight of the head forward, complicating the maintenance of erector spinae and core muscle strength, potentially leading to imbalances between the hip flexors and extensors [27]. To mitigate RRIs and maintain upper and lower body balance, the study recommends aligning the FHP with the average trunk lean angle of 6.19°.

This research also examined running styles concerning foot strike patterns. Of the 155 runners analyzed, 104 employed rearfoot strikes, and 32 used mid/forefoot strikes. The incidence of rearfoot strikes was 67% (105/155), while mid/forefoot strikes accounted for 23.9% (73/104). Despite more rearfoot strikers experiencing injuries compared to forefoot strikers, there was no significant difference between the normal and RRI groups. This suggests that RRIs occur regardless of foot strike type, indicating similar injury rates across different strike patterns. This result challenges previous research asserting that midfoot strikes result in fewer injuries [28]. The authors hypothesize that the overall posture of the knee, hip, and upper body during running exerts varying pressures on the ankle and foot, complicating the assertion that one strike type is superior. Macera et al. utilized a 3D stress method to measure pressure on different body parts among habitual rearfoot strike and mid/forefoot strike runners, finding higher pressure in mid/forefoot strikes during MPF and in rearfoot strikes during LR [29].

Inexperience in running is often linked to higher RRI rates. Many runners seeking clinical treatment are beginners or those who have sustained injuries after their first race. Reviews of existing studies indicate that runners with less than three years of experience are 2.2 times more likely to suffer from RRI compared to hobby runners with more than three years of experience [30]. This study corroborates these findings, showing that novice runners are more prone to RRI than recreational runners who run regularly (*p* = 0.033).

Evaluating the running posture of novice or recreational runners as suboptimal is inherently subjective, given that achieving a perfect posture in any sport is challenging. Nonetheless, a scientific approach enables us to infer injury-free running postures. This necessitates the use of equipment, such as the motion analysis system, which allows for efficient assessment using only a camera, thus eliminating the need for time-consuming tests and multiple sensor attachments. While this study did not encompass all factors, such as over-stride, the knee movement angle during touch down, contact time, ground reaction force, cadence, and running efficiency, it successfully identified a correlation between pelvic balance and spinal angle with running injuries. That is to say, it is anticipated that this streamlined evaluation method will become a valuable tool for swiftly assessing running posture and preventing injuries among a wide range of runners.

This study has several limitations. Firstly, the RRI and normal runner groups were retrospectively distinguished and compared. Secondly, the motion analysis system used only a rearview camera without sensors, potentially leading to measurement variability due to environmental factors, the runner’s attire, and accessories. Accordingly, the accuracy of measurements may be limited compared to systems with directly attached sensors. Future studies for reliability will be required to strengthen our study. However, as presented in Table 6, this instrument presents a more than acceptable range of reliability for measured outcomes in preliminary data. Thirdly, while associations between FHP, hip hike differences, and RRI were identified, specific types of RRIs could not be evaluated. Therefore, future studies including additional elements such as over-stride and knee movement angle are essential to provide a more comprehensive understanding of RRIs. Lastly, the nature of this study precludes establishing causal relationships between these variables and RRIs.

## 5. Conclusions

This study demonstrated that using a rear-facing camera to analyze the running motions of runners with and without RRIs is a convenient and effective method. Significant differences were observed in upper-body posture, center of gravity, and pelvic movement. Notably, the differences in hip hike and FHP emerged as key predictors of running-related musculoskeletal injury occurrence. Additionally, novice runners were found to be more prone to injuries. However, there was no significant difference in the type of strike patterns between runners with RRIs and the normal group. The use of a rear-facing camera provided a simple yet powerful tool for identifying these differences without relying on sensors. This method can aid in understanding factors associated with RRIs, offering valuable insights for rehabilitation, muscle strength enhancement, and injury prevention.

## Figures and Tables

**Table 1:** Demographics and running analysis measurements in this study.

Variables (n = 155)	Mean	SD	Min	Max	Cohen D
Mean age (years)	38.97	8.90	15	62	8.88
Weight (kg)	66.57	10.37	41.0	98.0	10.32
Height (cm)	174.04	44.55	150	717	44.56
Body mass index (kg/m^2^)	22.77	2.26	17.1	29.6	2.26
Running distance (km/week)	33.32	19.15	5	105	19.11
FHP (turtle neck, °)	8.19	4.51	−6	24	4.12
Trunk lean (°)	6.19	2.68	0	15	2.66
Hip rotation RT (°)	7.79	1.52	4	12	1.52
Hip rotation LT (°)	5.41	1.50	2	11	1.50
Horizontal movement of COG RT (cm)	1.69	1.70	0	7	1.69
Horizontal movement of COG LT (cm)	2.46	1.70	0	9	1.68
Vertical movement of COG (cm)	2.78	1.18	0	4	1.18
Pelvic rotation RT (°)	5.84	2.59	0	14	2.58
Pelvic rotation LT (°)	7.97	2.64	1	15	2.65
Hip hike RT (°)	3.08	1.17	1	5	0.87
Hip hike LT (°)	0.74	0.58	0	2	0.53
Difference of hip rotation (°)	2.37	1.41	−1	6	1.41
Difference of horizontal movement of COG (cm)	−0.77	3.09	−9	7	3.06
Difference of pelvic rotation (°)	−2.12	2.98	−8	7	2.96
Difference of hip hike (cm)	2.34	1.52	−1	5	1.13

SD, standard deviation; FHP, forward head posture; RT, right; LT, left; COG, center of gravity. The difference was calculated as RT-LT.

**Table 2:** Comparison of the demographics and running analysis measurements between the two groups in this study.

Variables (n = 155)	Normal (n = 50)	RRI (n = 105)	t	*p*
Sex (n, Male/Female)	33:17	77:45	N/A	0.347 *
Mean age (years)	40.18 ± 9.56	38.39 ± 8.55	1.17	0.243
Weight (kg)	64.65 ± 9.90	67.49 ± 10.50	−1.60	0.111
Height (cm)	169.12 ± 8.10	176.38 ± 53.76	−0.95	0.344
Body mass index (kg/m^2^)	22.51 ± 2.12	22.90 ± 2.32	−0.98	0.327
Running distance (km/week)	36.16 ± 20.93	31.97 ± 18.19	1.28	0.204
FHP (turtle neck, °)	5.50 ± 2.96	9.47 ± 4.56	−6.49	<0.001
Trunk lean (°)	5.58 ± 2.16	6.48 ± 2.86	−1.96	0.051
Hip rotation RT (°)	7.88 ± 1.44	7.74 ± 1.56	0.53	0.600
Hip rotation LT (°)	5.54 ± 1.54	5.35 ± 1.48	0.73	0.468
Horizontal movement of COG RT (cm)	1.38 ± 1.67	1.84 ± 1.70	−1.58	0.116
Horizontal movement of COG LT (cm)	2.88 ± 1.93	2.27 ± 1.55	2.13	0.035
Vertical movement of COG (cm)	2.78 ± 1.09	2.78 ± 1.23	−0.01	0.996
Pelvic rotation RT (°)	6.24 ± 2.72	5.65 ± 2.52	1.34	0.184
Pelvic rotation LT (°)	7.78 ± 3.01	8.06 ± 2.46	−0.61	0.544
Hip hike RT (°)	1.96 ± 0.57	3.61 ± 0.99	−13.15	<0.001
Hip hike LT (°)	1.10 ± 0.54	0.57 ± 0.52	5.75	<0.001
Difference of hip rotation (°)	2.34 ± 1.47	2.39 ± 1.39	−0.21	0.836
Difference of horizontal movement of COG (cm)	−1.50 ± 3.14	−0.43 ± 3.03	−2.04	0.043
Difference of pelvic rotation (°)	−1.54 ± 3.27	−2.41 ± 2.81	1.71	0.090
Difference of hip hike (°)	0.86 ± 0.64	3.04 ± 1.29	−14.03	<0.001

SD, standard deviation; FHP, forward head posture; RT, right; LT, left; COG, center of gravity. The difference was calculated as RT-LT. *p* and * *p* values were respectively calculated by the independence t-test and Chi-square test.

**Table 3:** The comparison of the types of strike between the two groups in this study.

Variables	Type of Strike		*p*
	Rearfoot	Midfoot + Forefoot	
Normal (n = 50)	31 (62.0%)	19 (38.0%)	0.351
RRI (n = 105)	73 (69.5%)	32 (30.5%)	
Total (n = 155)	104 (67.1%)	51 (32.9%)	

**Table 4:** Relationship between running experience and RRI.

Variables	Elite + Pro	Beginner	Recreational	*p*
Normal (n = 50)	3 (6.0%)	10 (20.0%)	37 (74.0%)	0.033 *
RRI (n = 105)	1 (1.0%)	37 (35.2%)	67 (63.8%)	
Total (n = 155)	4	47 (30.3%)	104 (67.1%)	

* *p* was calculated using Fisher’s exact test, which compared the beginner and recreational groups.

**Table 5:** Logistic regression analysis for RRI.

Independent Variables	B	SE	*p*	Exp (B)	95% CI
FHP (turtle neck, °)	0.42	0.11	<0.001	1.52	1.22–1.89
Trunk lean (°)	−0.07	0.17	0.703	0.94	0.67–1.31
Difference of hip rotation (°)	0.14	0.28	0.604	1.16	0.67–1.99
Difference of horizontal movement of COG (cm)	0.03	0.12	0.805	1.03	0.81–1.32
Vertical movement of COG (cm)	0.09	0.30	0.778	1.09	0.60–1.97
Difference of pelvic rotation (°)	−0.05	0.12	0.707	0.96	0.76–1.21
Difference of hip hike (°)	2.84	0.59	<0.001	17.17	5.40–54.62

SD, standard deviation; FHP, forward head posture; RT, right; LT, left; COG, center of gravity; CI, confidence interval. The difference was calculated as RT-LT.

**Table 6:** The intraclass correlation coefficient for measured outcomes using a motion analysis system (30 Hz, Exbody, Republic of Korea) with a rear-facing camera without a sensor.

Variables (n = 15)	ICC	*p*	Interpretation
FHP (turtle neck, °)	0.73	0.009	Acceptable reliability
Trunk lean (°)	0.70	0.016	Acceptable reliability
Hip rotation RT (°)	0.89	<0.001	Good reliability
Hip rotation LT (°)	0.93	<0.001	Excellent reliability
Horizontal movement of COG RT (cm)	0.63	0.038	Questionable reliability
Horizontal movement of COG LT (cm)	0.64	0.035	Questionable reliability
Vertical movement of COG (cm)	0.70	0.016	Acceptable reliability
Pelvic rotation RT (°)	0.94	<0.001	Excellent reliability
Pelvic rotation LT (°)	0.81	0.002	Good reliability
Hip hike RT (°)	0.87	<0.001	Good reliability
Hip hike LT (°)	0.88	<0.001	Good reliability

*p* was calculated using Fisher’s exact test, which compared the beginner and recreational groups.

## Data Availability

Data collected for this study, including individual patient data, will not be made available.
